# *Clitoria ternatea* as a phytopharmacological resource: plant metabolites, mechanistic evidence, translational prospects, and safety considerations

**DOI:** 10.3389/fphar.2026.1922024

**Published:** 2026-10-16

**Authors:** Dharmasoth Rama Devi, Salapu Neelima Maheswari, Rajana Krishna Saranya, Surisetty Aashritha, Nooka Gouri Sankar, Nissi Abigail Gurrala, Prithviraj Karak, Sayandeep K. Das

**Affiliations:** 1 Department of Pharmacognosy, Vignan Institute of Pharmaceutical Technology, Visakhapatnam, Andhra Pradesh, India; 2 Department of Physiology, Bankura Christian College, Bankura, India; 3 Department of Pathology, Shri B. M. Patil Medical College Hospital and Research Centre, BLDE (Deemed to be University), Vijayapura, Karnataka, India

**Keywords:** bioavailability, botanical drug development, *Clitoria ternatea*, cyclotides, evidence hierarchy, safety, ternatins

## Abstract

*Clitoria ternatea* L. (Fabaceae; butterfly pea) is used in traditional medicine and is increasingly developed as a food colorant, functional beverage, botanical preparation, and source of peptide scaffolds. Previous reviews have separately emphasized ethnomedicine, flower extraction, anthocyanins, or cyclotides; however, the evidence has rarely been integrated according to preparation identity, study design, bioavailability, safety, and translational readiness. This structured critical narrative review updates the literature through 8 August 2026. PubMed, Scopus, ScienceDirect, and Google Scholar were searched using species-name and domain-specific terms. Primary studies were prioritized, and evidence was stratified as chemical/analytical, *in silico*, isolated-target or cell-based, animal, and human. Study interpretation considered taxonomic authentication, plant part, extraction and chemical characterization, dose, controls, duration, model relevance, adverse-event reporting, and exposure plausibility. The principal metabolite classes are ternatin anthocyanins, flavonol glycosides, phenolic acids, triterpenoids, sterols, and cyclotides. Chemical assays such as DPPH and ABTS support redox characterization but do not establish pharmacological or therapeutic efficacy. Neurobehavioral, anti-inflammatory, metabolic, antimicrobial, organ-protective, and cytotoxic signals are reported mainly in preclinical models, often using incompletely standardized extracts. Human evidence is restricted to small acute crossover studies and exploratory adjunctive studies, including a 2026 study of flower tea in people with type 2 diabetes and diabetic distress. Intact ternatins are orally absorbed in rats, but systemic exposure is modest and human pharmacokinetic data are unavailable; metabolite exposure, formulation effects, chronic toxicity, reproductive safety, cyclotide immunogenicity, and botanical-drug interactions remain insufficiently defined. Recent genomic and peptide studies strengthen the biotechnology relevance of cyclotides and butelase-1 but should not be conflated with the efficacy of crude botanical preparations. Overall, *C. ternatea* is a promising source of defined food, botanical, and peptide products, but therapeutic claims require authenticated materials, marker-based standardization, exposure-linked pharmacology, and adequately controlled clinical trials.

## Graphical Abstract

**Figure:**
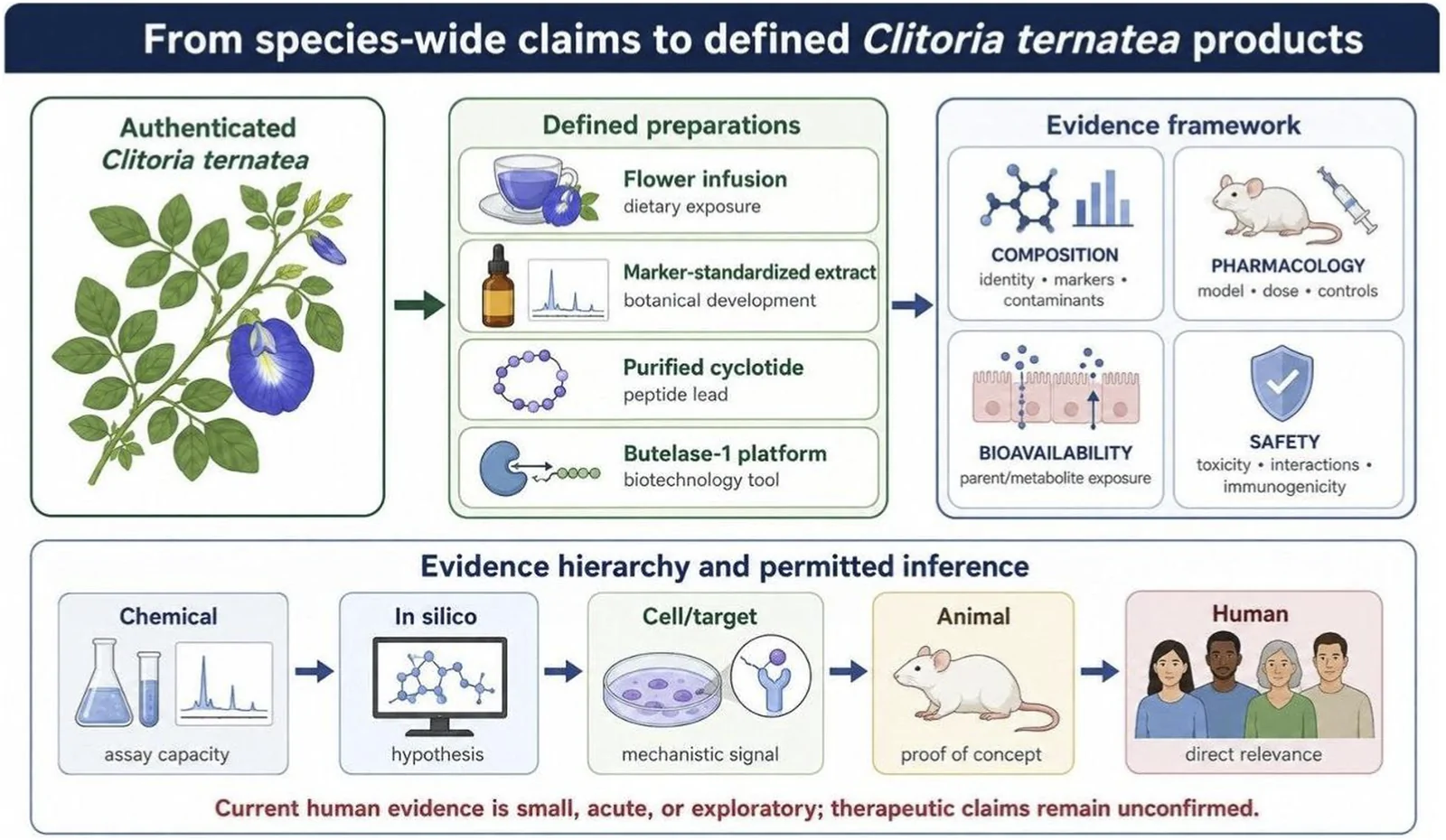

## Highlights

- Critically appraises *Clitoria ternatea* evidence by preparation and evidence level.
- Separates chemical antioxidant assays from cell, animal, and human pharmacology.
- Integrates ternatin bioavailability, cyclotide genomics, and butelase-1 biology.
- Finds human evidence limited to small acute or exploratory clinical studies.
- Defines standardization, safety, interaction, and clinical-development priorities.

## 1 Introduction

Plant-derived natural products remain important sources of chemical diversity for drug discovery, but biological activity in a screening assay is not equivalent to clinical efficacy. Contemporary phytopharmacology therefore requires authenticated botanical material, reproducible preparation and chemical characterization, pharmacologically relevant models, exposure-linked dose interpretation, and a balanced evaluation of efficacy and safety (Atanasov et al., 2021; Heinrich et al., 2020; Heinrich et al., 2022).

*Clitoria ternatea* L. (Fabaceae), commonly known as butterfly pea, is a perennial climber used in Ayurveda and several Southeast Asian traditional practices. Its flowers are also consumed as infusions and natural colorants. The roots, leaves, flowers, and seeds have distinct chemical profiles; consequently, evidence obtained with one plant part or preparation cannot automatically be transferred to another.

The species contains polyacylated delphinidin anthocyanins (ternatins), flavonol glycosides, phenolic acids, triterpenoids, sterols, lectins, and an unusually rich repertoire of cyclic cystine-knot peptides. These metabolites support applications ranging from pigment and food science to experimental pharmacology and peptide engineering (Kazuma et al., 2003a; Kazuma et al., 2003b; Oguis et al., 2019; Escher et al., 2020).

The canonical review by Mukherjee et al. (2008) established the ethnopharmacological and early experimental foundation for the species. Oguis et al. (2019) focused on cyclotides, Jeyaraj et al. (2021) on flower extraction and biological assays, and Vidana Gamage et al. (2021) on anthocyanin biosynthesis, stability, and applications. These reviews remain foundational, but none provides a product-specific transition analysis linking preparation identity, evidence hierarchy, exposure, safety, and regulatory readiness. The present review therefore extends rather than merely recapitulates their scopes by integrating 2024–2026 genomic, metabolomic, neuroprotective, formulation, bioavailability, and human evidence within the pharmacology-composition-bioavailability-safety framework.

The objectives were to: (i) distinguish preparation-specific evidence from species-wide claims; (ii) evaluate pharmacological studies according to model, dose, controls, duration, chemical definition, and translational relevance; (iii) separate chemical antioxidant assays from cell, animal, and human evidence; (iv) examine ternatin and cyclotide bioavailability, safety, and interaction risks; and (v) define product-specific priorities for clinical and regulatory development.

A structured search was conducted in PubMed, Scopus, ScienceDirect, and Google Scholar from database inception to 8 August 2026, with a final targeted rerun on 8 August 2026. ClinicalTrials.gov (2026) was searched for registered interventional studies. The core query combined (“*Clitoria ternatea*” OR “butterfly pea”) with terms covering metabolites, phytochemistry, extraction, anthocyanins, ternatins, cyclotides, pharmacology, mechanisms, bioavailability, pharmacokinetics, formulations, toxicity, safety, interactions, and clinical studies. Reference lists of the four foundational reviews were screened, and forward searches were used to identify recent primary studies. Full database-specific strings, eligibility criteria, and the data-extraction framework are provided in Supplementary Methods.

Peer-reviewed primary studies directly examining *C. ternatea* material or isolated *C. ternatea* metabolites were prioritized. Reviews were retained for historical context or broad synthesis but were not used as the sole support for specific pharmacological claims when primary evidence was available. Evidence was stratified as chemical/analytical assay, *in silico* prediction, isolated-target or cell-based study, animal study, and human study. Appraisal considered taxonomic authentication and voucher reporting, plant part and processing, extraction yield and marker characterization, dose and exposure, positive and negative controls, randomization and blinding, duration, model relevance, effect-size reporting, and adverse-event assessment. Because study designs and preparations were highly heterogeneous and reporting was often insufficient for a single validated risk-of-bias instrument across all evidence types, appraisal was domain-based rather than converted into an unsupported numerical quality score. Missing methodological details were recorded as not reported and were not inferred. No meta-analysis was attempted because preparation, model, and outcome heterogeneity precluded meaningful pooling.

## 2 Botanical identity, taxonomy, and quality assurance

The accepted name is *C. ternatea* L. (Fabaceae; subfamily Faboideae), order Fabales. The taxonomic table below is retained as a concise identity reference. For pharmacological work, the accepted scientific name, author citation, plant part, cultivar or flower colour, collection locality and date, authenticator, and voucher specimen should be reported; common names alone are insufficient (Royal Botanic Gardens, Kew, 2026; Heinrich et al., 2022) (Table 1).

**TABLE 1:** Taxonomic classification of *Clitoria ternatea* L.

Taxonomic rank	Classification
Kingdom	Plantae
Subkingdom	Viridiplantae
Division	Tracheophyta
Class	Magnoliopsida
Order	Fabales
Family	Fabaceae
Subfamily	Faboideae
Genus	*Clitoria* L.
Species	*Clitoria ternatea* L.

Because the chemical composition differs among flowers, roots, leaves, and seeds, botanical authentication is not a descriptive formality but a prerequisite for reproducible pharmacology and safety assessment.

### 2.1 Vernacular names, distribution, and diagnostic morphology

Common names include butterfly pea, blue pea, Aparajita, Shankhpushpi, and Shankupushpam. These names vary by region and may be shared with other species; therefore, they should never replace the binomial and voucher information in experimental reports.

#### 2.1.1 Distribution, cultivation, and macroscopic features

*Clitoria ternatea* is widely cultivated and naturalized across tropical and subtropical Asia, Africa, Australia, the Pacific, and the Americas. It grows in warm conditions on well-drained soils and is used as an ornamental, forage, cover crop, and nitrogen-fixing legume (Cook et al., 2005; Royal Botanic Gardens, Kew, 2026). The species is commonly propagated by seed. Agronomic conditions, genotype, flower colour, harvest stage, drying, and storage can alter the abundance and stability of pigments and other metabolites; these variables should be documented when material is intended for analytical or pharmacological comparison.

Mature plants are twining perennial climbers, generally reaching approximately 0.5–3 m depending on support and growing conditions. Young stems may be pubescent, whereas older basal portions become more lignified. The root system comprises a taproot with lateral roots and nitrogen-fixing nodules. Roots used for botanical preparations should be distinguished from aerial material because their triterpenoid, alkaloid, and peptide profile differs from that of flowers and leaves.

Leaves are alternate and imparipinnate, usually with five to seven elliptic to ovate leaflets. Leaf morphology, epidermal characters, and venation contribute to authentication but do not replace a voucher specimen or chemical fingerprint. Flowers are solitary or paired, axillary, papilionaceous, bisexual, and zygomorphic. Blue-flowered and white-flowered forms occur; blue petals contain the characteristic ternatin pigments. Flower colour and cultivar should be reported because they influence metabolite composition. The fruit is a flattened linear legume, commonly approximately 4–13 cm long, that becomes brown and dehisces at maturity. Pods generally contain several reniform brown to black seeds. Seeds are chemically and pharmacologically distinct from petals and roots, with proteins, lectins, lipids, and cyclic peptides that require preparation-specific characterization.

Macroscopic identity should be supported by collection metadata, comparison with an authenticated reference, and, where feasible, DNA-based or chromatographic confirmation. This is particularly important for commercial powders in which diagnostic morphology is reduced. The retained botanical schematic summarizes taxonomic placement and the macroscopic features most relevant to material identity (Figure 1). These features provide a practical quality-assurance bridge between field collection, pharmacognostic authentication, and downstream analytical standardization.

**FIGURE 1:**
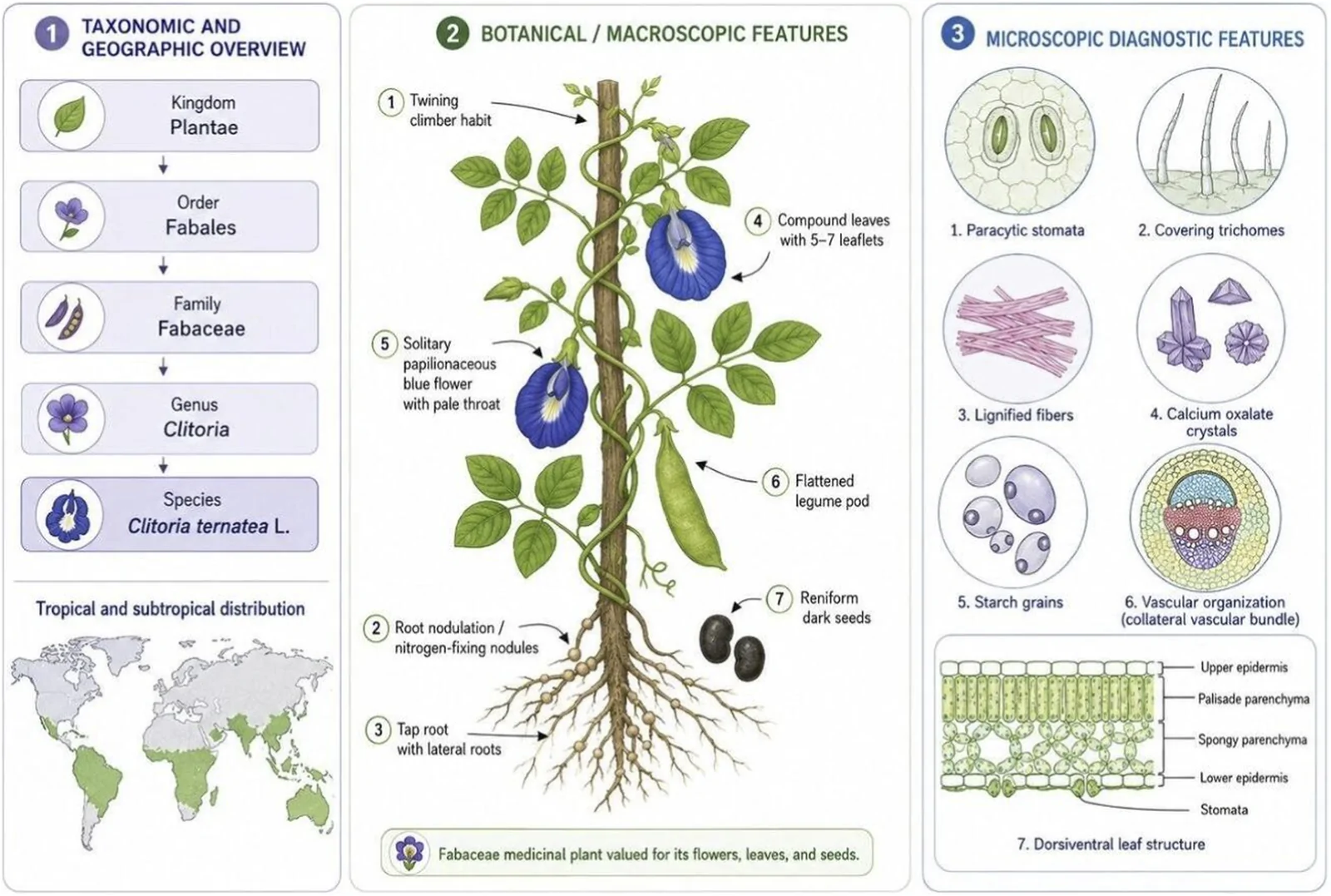
Botanical, macroscopic, and microscopic authentication profile of *Clitoria ternatea* L. The schematic summarizes accepted taxonomic placement, geographic distribution, growth habit, root nodulation, compound leaves, papilionaceous flowers, pods, seeds, and selected microscopic quality-control features. It is an original schematic created for this manuscript and finalized using Python (Pillow); no published figure was reproduced. Abbreviations: T.S., transverse section.

### 2.2 Botanical authentication as pharmacological quality control

Microscopy is summarized here only for its quality-control value. Diagnostic findings include a dorsiventral leaf with predominantly paracytic stomata, covering trichomes, collateral vascular tissue, lignified fibres, calcium oxalate crystals, and starch grains in powdered material. Stem and root sections show the expected vascular organization and secondary growth of a leguminous climber. These observations can detect substitution or adulteration, but a pharmacology-focused study should additionally report the voucher, source, processing, chemical fingerprint, marker content, and contaminant limits (Evans, 2009; Khandelwal, 2015; Heinrich et al., 2022). Figure 1 integrates the minimum botanical and microscopic features relevant to authentication (Figure 1).

## 3 Traditional and ethnomedicinal uses

*Clitoria ternatea* has a long history of use in Ayurveda and several Southeast Asian traditions. Ayurvedic sources classify it among Medhya Rasayana preparations and describe uses related to memory, anxiety, epilepsy, inflammation, respiratory symptoms, and general rejuvenation. These records are important for hypothesis generation but should be presented as traditional-use evidence rather than proof of efficacy (Mukherjee et al., 2008).

Roots, leaves, seeds, and flowers have been prepared differently for purgative, diuretic, respiratory, dermatological, neurological, and wound-related uses. The diversity of plant parts and preparation methods is a major source of pharmacological heterogeneity, and traditional claims cannot be transferred to a chemically unrelated extract without direct testing.

### 3.1 Southeast Asian food and traditional-use contexts

In Thailand, Malaysia, Indonesia, Vietnam, and the Philippines, the blue flowers are commonly used in infusions, beverages, rice, desserts, and topical preparations. Reported traditional wellness uses include cooling beverages, skin and hair care, and relief of minor inflammatory or menstrual symptoms. These practices indicate customary exposure, not validated treatment effects, and chronic-dose safety remains incompletely studied (Oguis et al., 2019; Vidana Gamage et al., 2021).

### 3.2 Nutritional and functional-food uses

The flowers are valued as pH-responsive natural colourants because of their ternatin anthocyanins. Food uses are the most mature application of the species, whereas disease-prevention claims require evidence beyond pigment stability or chemical antioxidant assays.

Flower infusions and food products provide acute dietary exposure to anthocyanins and flavonol glycosides. Small human crossover studies have examined postprandial physiological responses, but they do not establish long-term prevention or treatment of metabolic disease (Chusak et al., 2018; Thilavech et al., 2021).

Seeds and leaves contain proteins and other nutrients, but they are compositionally distinct from petals and should not be assumed to share the same safety or pharmacokinetic profile.

Accordingly, the review separates customary food exposure, concentrated botanical preparations, and isolated peptide products throughout the pharmacological and safety appraisal.

## 4 Plant metabolites and chemical standardization

Flowers, leaves, roots, and seeds contain different primary and specialized metabolites. Reliable comparison requires chromatographic or spectrometric definition of the preparation rather than qualitative phytochemical screening alone (Figure 2). The major classes are summarized below and in Tables 2, 3.

**FIGURE 2:**
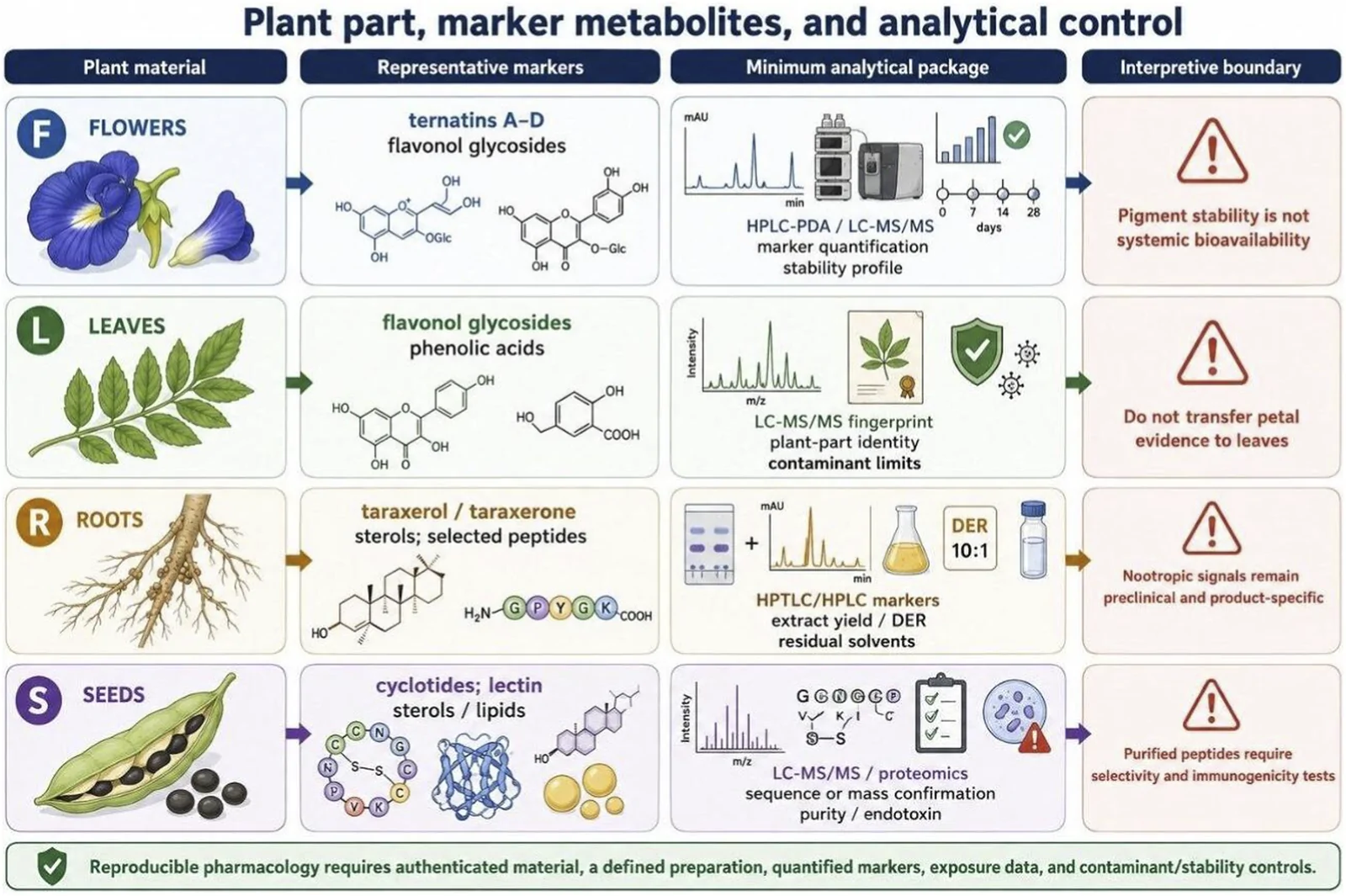
Phytochemical architecture and marker metabolites of *Clitoria ternatea*. The figure organizes metabolite and peptide categories by plant part and representative markers, including flavonol glycosides, ternatin anthocyanins, phenolic acids, alkaloids, glycosides, triterpenoids, sterols, saponins, cyclotides, lectins, and other historically reported seed peptides, while displaying butelase-1 separately as an enzyme/protein and indicating analytical approaches such as HPLC, LC-MS/MS, NMR, HPTLC, and proteomics (Kazuma et al., 2003a; Kazuma et al., 2003b; Neda et al., 2013; Escher et al., 2020; Oguis et al., 2019).

**TABLE 2:** Representative structures of primary-source-verified *Clitoria ternatea* constituents.

No.	Metabolite	Structures
1	Kaempferol 3-O-glucoside (petals; Kazuma et al., 2003a)	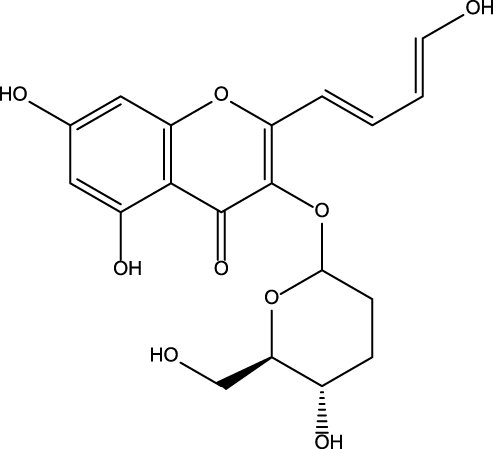
2	Kaempferol-3-O-rutinoside (petals; Kazuma et al., 2003a; b)	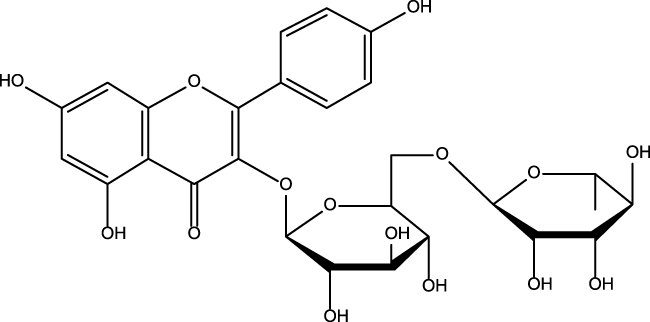
3	Quercetin (petals; Kazuma et al., 2003a)	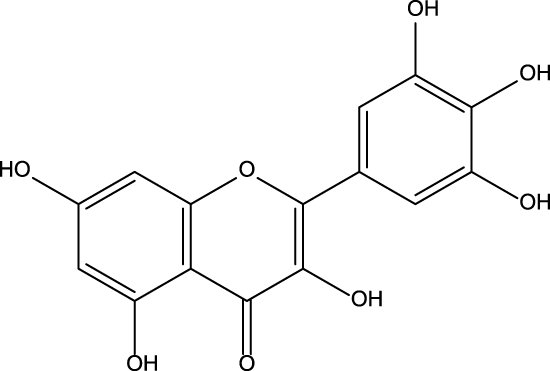
4	Myricetin (Kazuma et al., 2003a)	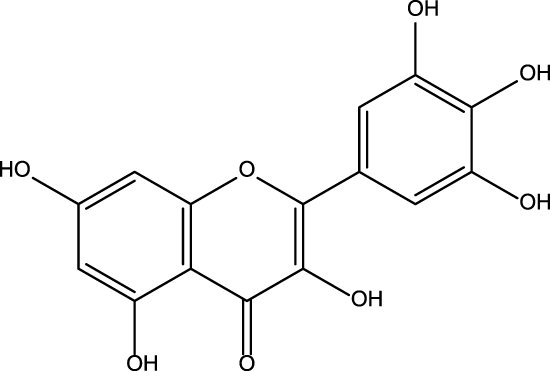
5	Epicatechin	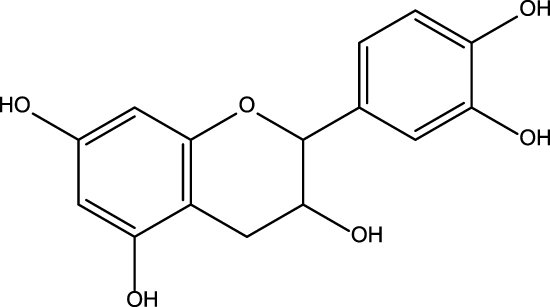
6	Taraxerol (root; Kumar et al., 2008)	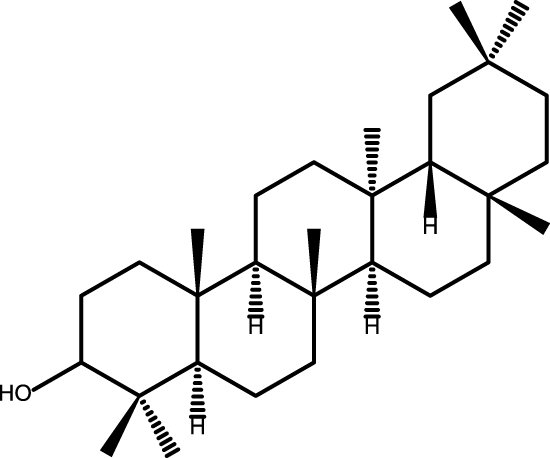
7	p-Coumaric acid (ternatin acyl moiety; Terahara et al., 1996; 1998)	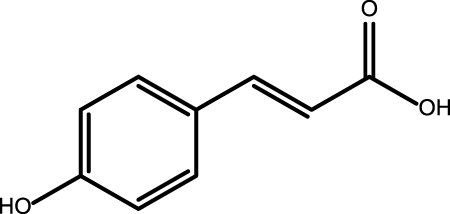

Only structure-defined constituents supported by primary isolation or validated analytical studies are shown. Generic ‘flavonoid monoglucoside’ and ‘anthoxanthin glycoside’ entries, free aglycone scaffolds not demonstrated as native constituents, and historically reported compounds without adequate structure-level documentation were removed. The p-coumaric acid structure represents the acyl unit incorporated into ternatins rather than evidence of free p-coumaric acid.

**TABLE 3:** Major *Clitoria ternatea* metabolite, peptide, and protein categories, analytical methods, and evidence-supported relevance.

Chemical/biological category	Representative metabolites	Plant part	Analytical method	Evidence-supported relevance	Primary references
Flavonol glycosides	Kaempferol-, quercetin-, and myricetin glycosides	Flowers; leaves	HPLC-PDA; LC-MS/MS; NMR	Identity markers; chemical redox and selected cell-based effects; exposure depends on glycoside form	Kazuma et al., 2003a, Kazuma et al., 2003b; Escher et al., 2020
Ternatin anthocyanins	Ternatins A-D and preternatins	Blue flowers	HPLC-PDA; LC-MS/MS; NMR	Characteristic pigments and formulation markers; intact oral absorption is demonstrated in rats, systemic exposure is low, and human pharmacokinetic data are unavailable	Terahara et al., 1996, Terahara et al., 1998; Ichiyanagi et al., 2021
Phenolic acids	p-Coumaric, ferulic, and related acids	Flowers; leaves	HPLC; UHPLC-QTOF-MS/MS	Part of multianalyte fingerprints; not species-specific as single markers	Escher et al., 2020
Tannins	Hydrolysable and condensed tannin signals	Leaves; roots	Spectrophotometry; LC methods	May contribute to protein binding and antimicrobial assays; interference controls required	Mukherjee et al., 2008
Triterpenoids	Taraxerol; taraxerone	Roots; aerial parts	HPTLC; LC-MS; GC-MS	Plant-part and solvent-dependent markers; oral exposure data are sparse	Kumar et al., 2008; Mukherjee et al., 2008
Sterols and lipids	β-sitosterol; long-chain alcohols	Seeds; roots	GC-MS; LC methods	Lipophilic fraction markers; pharmacology requires purified or standardized material	Mukherjee et al., 2008; Padmanabhan et al., 2024
Saponins	Saponin glycosides	Seeds; roots	LC-MS; validated colourimetric screening	Preliminary membrane-related hypotheses; metabolite-specific evidence is limited	Mukherjee et al., 2008
Cyclotides	Cliotides/Cter peptides	Multiple tissues	LC-MS/MS; proteomics; sequencing	Stable membrane-active peptide scaffolds; sequence-specific potency and safety	Nguyen et al., 2011, Nguyen et al., 2016; Oguis et al., 2019
Enzyme/protein: Butelase-1	Asx-specific peptide ligase	Plant tissue/enzyme preparation	Protein purification; activity assays	Biotechnology tool for macrocyclization; distinct from crude botanical exposure	Nguyen et al., 2014; Gilding et al., 2024
Lectins and other proteins	β-D-galactoside-specific lectin and other seed proteins	Seeds	Protein purification; electrophoresis; MS	Experimental protein/lectin evidence only; purity, selectivity, exposure, and immunogenicity require definition	Naeem et al., 2007; Oguis et al., 2019

Abbreviations: GC-MS, gas chromatography-mass spectrometry; HPLC-PDA, high-performance liquid chromatography with photodiode-array detection; HPTLC, high-performance thin-layer chromatography; LC-MS/MS, liquid chromatography-tandem mass spectrometry; NMR, nuclear magnetic resonance; UHPLC-QTOF-MS/MS, ultra-high-performance liquid chromatography-quadrupole time-of-flight tandem mass spectrometry.

### 4.1 Flavonol glycosides and ternatin anthocyanins

Kaempferol-, quercetin-, and myricetin-derived glycosides occur particularly in leaves and petals. Hydroxylation patterns influence redox and protein interactions, whereas glycosylation changes aqueous solubility, extraction, intestinal transport, and metabolism. Consequently, aglycone-equivalent reporting cannot substitute for identification and quantification of the native glycosides (Kazuma et al., 2003a; Kazuma et al., 2003b; Escher et al., 2020).

The blue colour of the petals is produced by ternatins, polyacylated delphinidin glycosides. Acylation and intramolecular co-pigmentation increase pigment stability. Despite their high molecular mass, intact ternatins have been detected in rat plasma after oral dosing; however, measured systemic concentrations were low and human pharmacokinetics remain unknown (Ichiyanagi et al., 2021). Ternatins A-D and related preternatins are therefore strong identity and quality markers, although systemic biological exposure may be dominated by degradation and conjugated metabolites (Terahara et al., 1996; Terahara et al., 1998; Ichiyanagi et al., 2021; Yu et al., 2024).

Recent UHPLC-QTOF-MS/MS and related profiling studies have expanded the chemical map of petals and seeds, but reported abundance remains sensitive to cultivar, colour line, harvest stage, drying, pH, solvent, and analytical method (Escher et al., 2020; Padmanabhan et al., 2024).

Ternatin-rich and quercetin-glycoside-rich fractions show different cell-based anti-inflammatory responses; this fraction specificity argues against treating total phenolic content or chemical radical-scavenging capacity as a universal mechanism (Nair et al., 2015).

### 4.2 Alkaloids and glycosides

Qualitative studies report nitrogen-containing metabolites and several glycoside classes in different organs, but many claims lack metabolite-level confirmation. These categories should be retained only when supported by authenticated material, a validated analytical signal, and, ideally, an isolated reference standard. Glycosylation may improve water extraction while simultaneously reducing passive permeability.

### 4.3 Phenolic acids and tannins

p-Coumaric, ferulic, and related phenolic acids are reported in flower and leaf profiles. Their smaller size may favour bioaccessibility relative to complex polyacylated pigments, but they are not species-specific and should form part of a multianalyte fingerprint rather than serve as sole identity markers.

Tannins can contribute to protein binding and antimicrobial assay effects, yet nonspecific precipitation can confound isolated-target experiments. Preparation concentration, assay interference controls, and normal-cell or host-toxicity testing are therefore essential.

### 4.4 Saponins, triterpenoids, and sterols

Saponins are reported in roots and seeds, but structure-specific pharmacology remains limited. Pentacyclic triterpenoids, including taraxerol and taraxerone, and sterols such as β-sitosterol are more efficiently recovered by nonpolar or hydroalcoholic extraction and require plant-part-specific standardization (Kumar et al., 2008; Mukherjee et al., 2008).

Lipophilicity may promote membrane interactions but does not establish oral bioavailability. Quantified markers, extraction yield, residual-solvent control, and pharmacokinetic assessment are necessary before a triterpenoid-rich fraction can be compared across studies.

### 4.5 Cyclotides and other peptides; butelase-1 as a peptide ligase

Cyclotides are head-to-tail cyclic peptides stabilized by a cystine-knot motif. Nguyen et al. (2011) identified 15 heat-stable cysteine-rich peptides, including 12 novel cliotides, and demonstrated that *C. ternatea* cyclotides arise from unusual chimeric albumin-1/cyclotide precursors. Transcriptomic profiling subsequently identified candidate genes implicated in cyclotide biosynthesis, although predicted precursors still require peptide-level confirmation (Kalmankar et al., 2020). These peptides occur across multiple tissues and exhibit membrane-active antimicrobial and cytotoxic properties, but potency and selectivity are sequence-specific.

Distinct from metabolite classes, butelase-1 is an Asx-specific ligase enzyme/protein isolated from *C. ternatea*, catalyses efficient peptide macrocyclization and is important for peptide engineering (Nguyen et al., 2014). More recent chromosome-level work showed expansion and diversification of albumin-1 genes together with neofunctionalized ligase-type asparaginyl endopeptidases, while a 2026 petal study broadened the tissue-level cyclotide inventory (Gilding et al., 2024; Ikeda et al., 2026). These biotechnology findings should not be interpreted as evidence that a crude flower infusion delivers an efficacious peptide dose.

A β-D-galactoside-specific seed lectin has been purified and structurally characterized (Naeem et al., 2007). Historical reports of “insulin-like” seed peptides remain insufficiently characterized and are not treated here as evidence of insulin-mimetic or antidiabetic activity. Peptide or lectin studies require sequence or mass confirmation, purity, endotoxin control, haemolysis and normal-cell selectivity testing, and preparation-specific immunogenicity assessment.

A 2024 UHPLC-QTOF-HRMS study of seeds demonstrated the value of organ-specific metabolomic profiling and further underscored that seed chemistry cannot be inferred from petal studies (Padmanabhan et al., 2024).

Table 2 presents representative chemical structures, and Table 3 links major chemical and biological categories with analytical approaches and evidence-supported relevance. These tables are descriptive chemistry resources; pharmacological inference is reserved for studies using defined preparations and biologically relevant models (Tables 2, 3).

### 4.6 Structure-activity and standardization implications

Polyacylation stabilizes ternatin colour but can reduce intact permeability; glycosylation alters solubility and metabolism; triterpenoid lipophilicity favours nonpolar extraction; and the cyclic cystine-knot architecture of cyclotides confers proteolytic stability while surface charge and hydrophobicity govern membrane activity. These relationships provide testable hypotheses, not automatic therapeutic attribution.

For every pharmacological or toxicological preparation, reporting should include accepted taxonomy, voucher or authenticated supplier, plant part, processing, solvent, drug-to-extract ratio, extraction yield, validated chromatographic fingerprint, quantified marker metabolites, stability, and contaminant limits in accordance with ConPhyMP principles (Heinrich et al., 2022).

## 5 Pharmacological evidence: hierarchy and critical appraisal

The evidence is interpreted by preparation and study design rather than as a single species-wide therapeutic profile. Chemical DPPH, ABTS, FRAP, and related assays are analytical measures; *in silico* docking is hypothesis-generating; cell and isolated-target studies provide mechanistic signals; animal studies provide proof of concept; and human studies provide the highest translational relevance. Across all levels, incomplete botanical authentication, extract standardization, dose reporting, control selection, and blinding frequently limit confidence.

### 5.1 Neurological and neurobehavioral evidence

Early rodent studies reported improvements in learning, memory, and central cholinergic activity after root or whole-plant preparations (Taranalli and Cheeramkuzhy, 2000; Rai et al., 2002; Jain et al., 2003). Later work reported anticholinesterase and cognitive effects with repeated root-extract dosing, and bio-enhanced fractions were evaluated in chronic cerebral hypoperfusion models (Damodaran et al., 2020; Ahad et al., 2023).

This is one of the better developed preclinical domains, but the preparations are not chemically interchangeable, most studies are short, and randomization, blinding, sample-size justification, brain exposure, and disease-modifying endpoints are inconsistently reported. A 2025 metabolite-profiled study strengthened chemical linkage but remains a single *in vitro*/*in vivo* programme without human confirmation (Chowdhury et al., 2025).

### 5.2 Metabolic and cardiometabolic evidence

Animal models report glucose- and lipid-related effects of flower, leaf, or mixed preparations, including changes in oxidative and inflammatory endpoints (Solanki and Jain, 2010; Wang et al., 2022; Widowati et al., 2023). These findings support biological plausibility but do not establish treatment of human diabetes or dyslipidaemia because extract composition, marker-normalized exposure, and model-to-human dose translation are incomplete.

Two small randomized crossover studies in men examined acute flower-extract exposure with sucrose or a high-fat meal. They reported selected postprandial glucose, insulin, triglyceride, free-fatty-acid, or redox changes, but used single exposures and surrogate endpoints; neither study demonstrated sustained glycaemic control, prevention of complications, or clinical benefit (Chusak et al., 2018; Thilavech et al., 2021).

### 5.3 Anti-inflammatory, analgesic, and respiratory evidence

Methanolic root extract reduced edema, nociception, and pyrexia in acute animal models at 200 and 400 mg/kg, and flower or ethanolic preparations have shown anti-arthritic and synovial-matrix signals (Devi et al., 2003; Adhikary et al., 2018; Swathi et al., 2021). In LPS-stimulated macrophages, chemically profiled petal fractions produced fraction-specific effects: quercetin glycosides and ternatin anthocyanins did not act identically, and some anti-inflammatory effects were not explained by ROS scavenging alone (Nair et al., 2015).

Root preparations have also been tested in acute bronchospasm, allergy, and cough models (Taur and Patil, 2011; Singh et al., 2018). These models support proof of concept but do not reproduce chronic airway inflammation, remodelling, or patient-level outcomes. Claims involving NF-κB, COX-2, or iNOS should therefore be restricted to preparations and models in which those targets were directly measured.

### 5.4 Antimicrobial, antifungal, and antiparasitic evidence

Plant-part extracts and cyclotide-rich preparations inhibit selected microorganisms *in vitro*, while a 2024 study combined flower profiling, *in silico* analysis, and *in vitro* testing for fungal keratitis-related organisms (Kamilla et al., 2009; Nguyen et al., 2016; Yolin Angel et al., 2024). Diffusion-zone data are strongly affected by solubility and diffusion and should not substitute for standardized minimum inhibitory concentrations, time-kill studies, host-cell selectivity, resistance testing, or *in vivo* infection models.

Larvicidal, anthelmintic, and other antiparasitic reports remain screening-level evidence. They may identify agricultural or lead-discovery applications but cannot be translated into human anti-infective use without defined active material, pharmacokinetics, and host safety.

### 5.5 Organ-protective and cytotoxic evidence

Leaf and root extracts have improved biochemical and histological endpoints in acute toxin-induced liver or kidney injury models (Nithianantham et al., 2011; Sarumathy et al., 2011). These experiments test injury attenuation rather than chronic liver or kidney disease, and efficacy is difficult to compare because the extracts, doses, and marker composition differ.

Flower extracts, anthocyanin-rich fractions, lectins, and cyclotides show concentration-dependent cytotoxicity in selected cell systems (Neda et al., 2013; Jeyaraj et al., 2022; Nguyen et al., 2011). This supports lead identification, not anticancer efficacy. Tumour selectivity, normal-cell toxicity, clinically achievable exposure, validated molecular targets, and *in vivo* efficacy remain insufficiently defined.

### 5.6 Human clinical evidence

Human evidence remains limited to small studies of defined or semi-defined flower preparations. The acute randomized crossover studies by Chusak et al. (2018) and Thilavech et al. (2021) addressed postprandial physiology. Jassanti et al. (2025) reported a prospective randomized, single-blind, multicentre, active-control study. The article states that 30 participants were assigned (15 per group) and 28 were analysed after two exclusions, whereas the ClinicalTrials.gov (2026) record (NCT06794502) lists an actual enrollment of 28. The registry record was first posted on 27 January 2025, after the May–July 2024 study period and after the article was submitted on 21 October 2024; the registration was therefore retrospective. Participants received a 40 mL/day decoction prepared from five dried flowers or control in addition to standard rifampicin, isoniazid, pyrazinamide, and ethambutol therapy for 2 months. The evidence is preliminary and exploratory: the between-group IFN-γ result was based on a change-factor analysis in a very small sample and is not a validated surrogate for clinical benefit; AFB findings were described rather than tested inferentially, and clinical symptoms did not differ significantly between groups. The absence of placebo control, unclear allocation concealment, inconsistent masking descriptions between the paper and registry, limited chemical characterization, incomplete adverse-event ascertainment, and no anti-TB-drug or botanical-marker pharmacokinetics preclude conclusions about efficacy or interaction safety.

Mani et al. (2026) reported a 12-week comparative study using decoctions prepared from 5 or 10 g of authenticated flowers (voucher 78/2021), administered twice daily with metformin. The article describes four groups of 30 but gives internally inconsistent participant accounting (114 initially enrolled, 90 in the final analysis, and separate healthy-control and diabetic cohorts). Reported changes in Diabetes Distress Scale-17, serum protein carbonyls, and urinary 5-hydroxyindoleacetic acid are therefore exploratory. The absence of a placebo, unclear randomization, blinding, and attrition reporting, lack of marker standardization, and absence of metformin or botanical-marker pharmacokinetics preclude efficacy or interaction conclusions.

### 5.7 Overall evidence grade and research priorities

The most consistent conclusion is that *C. ternatea* contains preparations with measurable biological activity, not that the species is a clinically established treatment. Evidence certainty is highest for chemical identity and pigment functionality, moderate-to-low for selected preclinical pharmacology, and very low for therapeutic efficacy in humans.

Future studies should pre-specify the product, authenticate the material, quantify markers, report extraction yield and dose, use appropriate positive and negative controls, randomization and blinding, establish concentration-response and exposure-response relationships, and incorporate adverse-event and interaction monitoring. Table 4 provides study-level details and limitations for representative primary studies (Table 4) (Figure 3).

**TABLE 4:** Critical appraisal of representative pharmacological and clinical studies of *Clitoria ternatea*.

Study/evidence level	Preparation and identity/chemistry	Model and controls	Dose/duration	Main finding and principal limitation	Primary source
Neurobehavioral; *in vivo*	Alcoholic aerial-part and root extracts; incompletely chemically characterized	Electroshock-induced amnesia in rats; vehicle and pyritinol comparator	300 or 500 mg/kg orally; acute experimental programme	Improved memory retention at 300 mg/kg, with root extract more active; no marker standardization, PK, or contemporary bias reporting	Taranalli and Cheeramkuzhy, 2000
CNS screening; *in vivo*	Methanolic extract; voucher reported	Multiple rodent CNS paradigms with model-specific controls	Approx. 30–400 mg/kg; acute	Anxiolytic/anticonvulsant signals; broad screening and limited bias reporting	Jain et al., 2003
Inflammation/pain; *in vivo*	Methanolic root extract	Edema, nociception, pyrexia; vehicle and standard drugs	200 and 400 mg/kg orally; acute	Reduced model endpoints; crude extract, no exposure or chronic assessment	Devi et al., 2003
Inflammation; cell/mechanistic	Chemically profiled petal fractions	LPS-stimulated macrophages; LPS and fraction comparisons	Concentration series; short cell exposure	Fraction-specific, partly ROS-independent effects; no human exposure linkage	Nair et al., 2015
Cognition/AChE; *in vivo*	Methanolic root extract; limited marker definition	Rodent cognition and AChE; vehicle/model and positive-comparator groups	100, 200, or 300 mg/kg orally for 28 days	Dose-related cognitive and AChE signal; no disease-modifying endpoint or human PK	Damodaran et al., 2020
Cerebral hypoperfusion; *in vivo*	HPLC-UV-standardized bio-enhanced root fraction containing approximately 25% (w/w) clitorienolactone A	Rat chronic cerebral hypoperfusion model; sham, model, vehicle, fraction, and clitorienolactone-A groups	Fraction 10, 20, or 40 mg/kg orally for 4 weeks; clitorienolactone A 10 mg/kg also evaluated	Behavioural and hippocampal-plasticity signal; exploratory single programme without PK or human confirmation	Ahad et al., 2023
Neuroprotection; *in vitro* + *in vivo*	Metabolite-profiled root/flower combination	Enzyme, behavioral, biochemical, and histology controls	200 mg/kg *in vivo*	Neurobehavioral protection; single programme without human confirmation	Chowdhury et al., 2025
Obesity/inflammation; *in vivo*	Blue-petal extract	High-fat/high-fructose mice; diet and intervention controls	0.25%, 0.5%, or 2% (w/w) in diet for 16 weeks	Improved selected metabolic/inflammatory endpoints; preventive model and uncertain human equivalence	Wang et al., 2022
Diabetes/dyslipidaemia; *in vivo*	Flower extract	Diabetic-dyslipidaemic rats; disease and comparator groups	200, 400, or 800 mg/kg orally for 28 days	Glucose/inflammation/GSK-3β changes; independent replication and standardized preparation needed	Widowati et al., 2023
Bronchospasm; *in vivo*	Root extract; incompletely chemically characterized	Guinea-pig histamine/acetylcholine challenge; standard comparator	100 or 150 mg/kg intraperitoneally; acute challenge	Prolonged pre-convulsive time; no chronic-airway, marker-exposure, or clinical evidence	Taur and Patil, 2011
Allergy/cough; *in vivo*	Root-derived preparation	Model-specific anti-allergic and antitussive controls	100, 200, or 400 mg/kg orally in acute model programmes	Reduced cough/allergy endpoints; preparation standardization and chronic models limited	Singh et al., 2018
Hepatoprotection; *in vivo*	Leaf extract	Paracetamol injury in mice; toxin, vehicle, comparator groups	200 mg/kg orally in an acute paracetamol-injury model	Biochemistry/histology improved; acute toxin model, not chronic disease	Nithianantham et al., 2011
Nephroprotection; *in vivo*	Ethanolic extract	Drug-induced renal injury; disease and treatment groups	*250 or 500 mg/kg orally for 14 days*	Renal markers/histology improved; extract chemistry and long-term function limited	Sarumathy et al., 2011
Antimicrobial; *in vitro*	Plant-part solvent extracts	Diffusion susceptibility tests; microbial/solvent controls	Extract-dependent concentration series; MIC/MBC/MFC values where reported	Growth inhibition; diffusion bias, variable chemistry, no *in vivo* PK	Kamilla et al., 2009
Antifungal keratitis; *in vitro* + *in silico*	Profiled flower extract	Fungal assays and target modelling; comparator/model controls	Concentration series	Antifungal signal; no ocular animal model, corneal PK, or clinical efficacy	Yolin Angel et al., 2024
Safety; *in vivo*	Extract; limited chemical definition	Mice with untreated control	500, 1,000, or 2000 mg/kg for 14 days	No mortality at 500–1,000 mg/kg; liver/kidney histopathological changes at 2000 mg/kg; short study and no chronic NOAEL	Khatib and Tofrizal, 2024
Postprandial glycaemia; human crossover	Voucher-authenticated aqueous flower extract (BKU066793); 1:20 w/v, 90 °C–95 °C for 4 h twice, spray-dried; total phenolics and anthocyanins reported	15 healthy men; randomized five-condition crossover	1 or 2 g extract with or without sucrose; single exposure; 3 h	Selected glucose, insulin, and redox changes; small male-only acute study with surrogate outcomes	Chusak et al., 2018
Postprandial lipaemia; human crossover	1 or 2 g flower extract with high-fat meal	16 overweight/obese men; randomized crossover	Single dose; 6 h	2 g altered triglyceride/free-fatty-acid responses; no clinical endpoint	Thilavech et al., 2021
Pulmonary tuberculosis adjunct; human exploratory	Decoction of five dried flowers boiled in 40 mL water at 100 °C for 10 min; total flavonoid and phenolic contents measured	Prospective randomized active-control study; article reports 30 assigned and 28 analysed, whereas NCT06794502 lists actual enrollment of 28; registration was first posted after study completion; standard rifampicin, isoniazid, pyrazinamide, and ethambutol therapy in both groups	40 mL/day for 2 months	Preliminary exploratory change-factor signal for IFN-γ; AFB findings were descriptive and clinical symptoms did not differ significantly; very small sample, retrospective registration, no placebo, inconsistent masking descriptions, limited chemical characterization, and no anti-TB-drug or botanical-marker PK	Jassanti et al., 2025
Diabetic distress; human exploratory	Fresh flower decoction: 5 or 10 g flowers boiled in 150 mL water to 100 mL; voucher 78/2021; administered with metformin; no marker-standardized fingerprint	Four-group comparative study (30/group stated): healthy control, metformin-only control, and two metformin-plus-tea groups; participant accounting is internally inconsistent (114 enrolled/90 final vs. four groups of 30)	5 or 10 g flowers per administration, twice daily after breakfast and dinner for 12 weeks (nominal 10 or 20 g/day)	DDS-17, serum protein carbonyl, and urinary 5-HIAA signals; no placebo; randomization, blinding, and attrition reporting inadequate; no botanical-marker or metformin pharmacokinetics	Mani et al., 2026

Abbreviations: AChE, acetylcholinesterase; AFB, acid-fast *bacillus*; DDI, drug-drug interaction; DDS-17, Diabetes Distress Scale-17; 5-HIAA, 5-hydroxyindoleacetic acid; GSK-3β, glycogen synthase kinase-3, β; LPS, lipopolysaccharide; PK, pharmacokinetics; ROS, reactive oxygen species. Study details were transcribed only when verifiable in the primary report; missing information was recorded as not reported and was not inferred.

**FIGURE 3:**
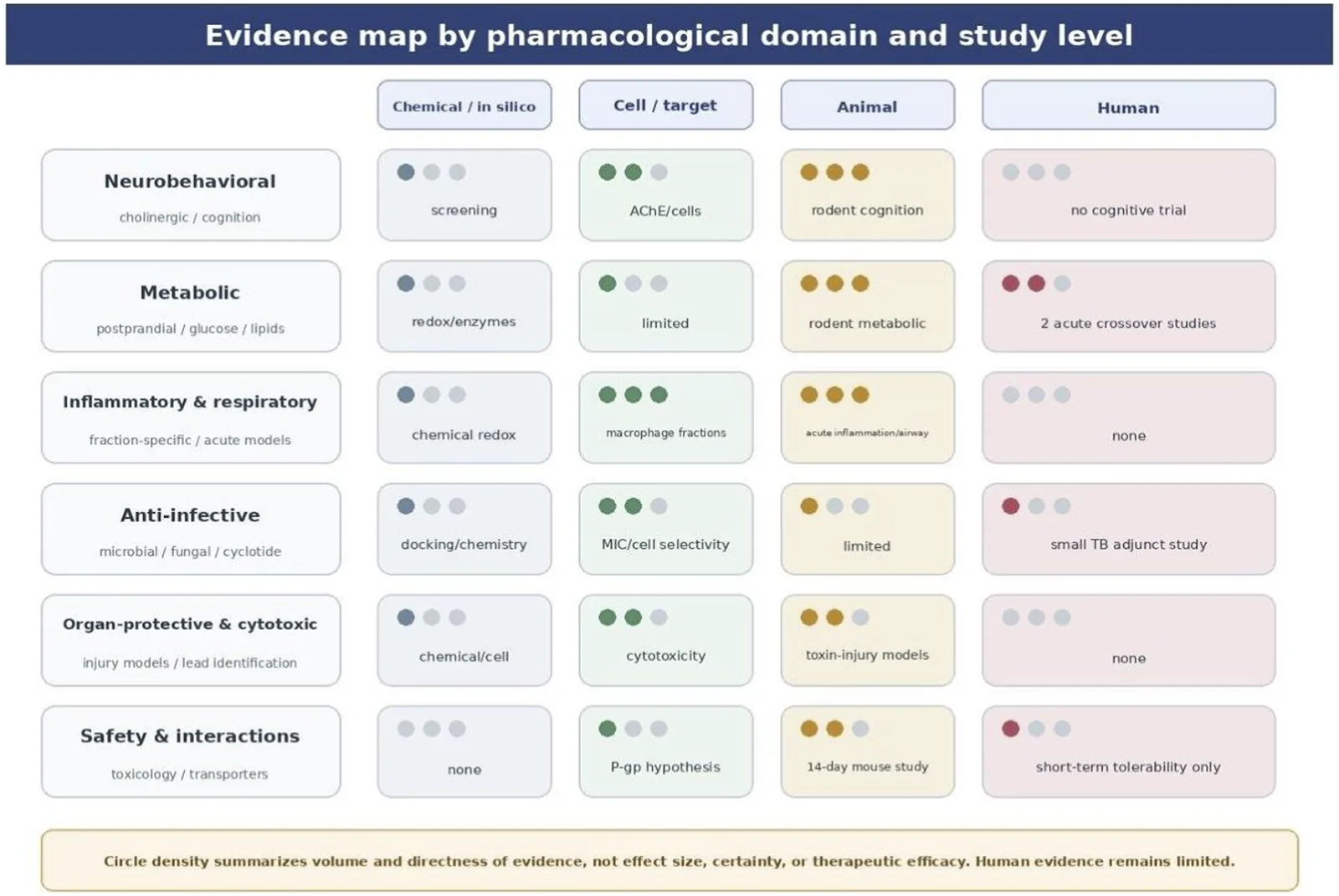
Evidence map of *Clitoria ternatea* preparations across reported activity domains. Chemical/analytical, *in silico*, cell-based, animal, traditional-use, and human evidence are distinguished, and the labels denote study endpoints rather than established treatment indications. Original schematic prepared by the authors using Python (Pillow).

## 6 Extraction, standardization, and manufacturing considerations

Extraction must be matched to the intended product and marker metabolites. Hot-water infusion is most relevant to customary flower-beverage exposure, whereas aqueous ethanol and other hydroalcoholic systems recover broader anthocyanin and flavonol profiles. Acidification, temperature, time, light, oxygen, drying, and storage can alter ternatin yield and degradation (Jeyaraj et al., 2021; Vidana Gamage et al., 2021).

Roots and seeds require separate optimization because their triterpenoids, sterols, proteins, lectins, and cyclotides differ from petal pigments. Peptide-focused workflows typically combine aqueous-organic extraction, solid-phase enrichment, reversed-phase chromatography, and mass spectrometry; these preparations are not equivalent to dietary infusions (Nguyen et al., 2011; Oguis et al., 2019).

Ultrasound- and microwave-assisted extraction can reduce time and solvent consumption, but scale-up requires control of temperature, cavitation or microwave energy distribution, solvent recovery, and batch-to-batch marker yield. Supercritical carbon dioxide is suited mainly to lipophilic fractions unless a polar modifier is added and remains insufficiently validated for *C. ternatea* pharmacology (Mehmood et al., 2019; Izirwan et al., 2020; Santos and Martins, 2023).

Industrial scalability should be evaluated with extraction yield, solvent and energy use, equipment geometry, process reproducibility, residual solvents, microbiological quality, pesticides, heavy metals, mycotoxins, stability, and cost. A higher total-phenolic yield or DPPH value alone is not an adequate optimization endpoint for a pharmacological product.

Standardization should combine an accepted botanical identity, plant-part and cultivar specification, drug-to-extract ratio, process parameters, validated chromatographic fingerprint, quantified markers, and contaminant limits. Bioactivity-guided fractionation can support causal inference but may also remove synergistic or antagonistic mixture effects; both the fraction and the parent preparation should therefore be characterized. Table 5 compares conventional and emerging extraction approaches according to their most appropriate material, target metabolites, evidence context, and scale-up limitations (Table 5).

**TABLE 5:** Extraction approaches, target metabolites, and standardization considerations for *Clitoria ternatea*.

Extraction approach	Solvent/process	Material or target	Main recovered metabolites	Evidence context	Scale or standardization issue	Primary references
Hot-water infusion/decoction	Water; controlled mass, time, temperature, pH	Flowers; dietary exposure	Ternatins; flavonol glycosides	Human beverage/food relevance	Marker dose and preparation variables must be reported	Chusak et al., 2018; Thilavech et al., 2021
Hydroalcoholic maceration	Water-ethanol mixtures	Flowers; leaves	Anthocyanins; flavonols; phenolic acids	Broad analytical and preclinical use	Drug-to-extract ratio, yield, solvent strength, and stability required	Jeyaraj et al., 2021
Soxhlet/solvent extraction	Petroleum ether, chloroform, methanol	Roots; seeds	Triterpenoids; sterols; alkaloid signals	Preclinical screening	Heat exposure and residual solvents; not representative of dietary use	Mukherjee et al., 2008; Kumar et al., 2008
Ultrasound-assisted extraction	Aqueous alcohol; controlled sonication	Flowers; phenolics/anthocyanins	Ternatins; flavonols	Process optimization	Scale-dependent cavitation and temperature control	Mehmood et al., 2019; Santos and Martins, 2023
Microwave-assisted extraction	Water-ethanol; controlled power	Flowers; leaves	Anthocyanins; flavonoids	Rapid process intensification	Local overheating and pigment-degradation risk	Izirwan et al., 2020
Supercritical-fluid extraction	Carbon dioxide with optional ethanol modifier	Seeds/roots; lipophilic fraction	Sterols; lipophilic metabolites	Emerging process concept	Poor polar-pigment recovery; limited species-specific pharmacology	Chemat et al., 2020; Jeyaraj et al., 2021
Liquid-liquid fractionation	Ethyl acetate, n-butanol, aqueous phases	Parent extracts	Polarity-defined fractions	Mechanistic and bioactivity comparison	Partition coefficients, recovery, and parent-extract linkage required	Nair et al., 2015; Jeyaraj et al., 2022
Preparative chromatography	Column or preparative LC gradients	Defined fractions/metabolites	Flavonols; triterpenoids; pigments	Isolation and structure-activity studies	Cost, throughput, purity, and loss of mixture interactions	Kazuma et al., 2003a, Kazuma et al., 2003b; Kumar et al., 2008
Peptide extraction/enrichment	Aqueous-organic extraction, SPE, RP-HPLC	Multiple tissues; cyclotides	Cliotides/Cter peptides	Peptide discovery and engineering	Sequence purity, recovery, oxidation state, endotoxin and selectivity testing	Nguyen et al., 2011; Oguis et al., 2019
Analytical HPTLC/HPLC	Validated mobile phases	Roots; flowers; finished extracts	Taraxerol; ternatins; flavonols	Fingerprinting and quantitative quality control	Not an extraction scale-up method; validation and reference standards required	Kumar et al., 2008; Makasana et al., 2023
Microencapsulation	Spray drying with food-compatible wall materials	Ternatin-rich flower material	Protected anthocyanins	Stability and food formulation	Improved stability is not proof of improved human bioavailability	Ab Rashid et al., 2021; Multisona et al., 2023
Enzyme/fermentation-assisted extraction	Aqueous bioprocessing	Flowers or plant matrix	Released phenolics and pigments	Emerging green approach	Enzyme specificity, microbial control, marker stability, and species-specific validation	Jeyaraj et al., 2021

Abbreviations: HPLC, high-performance liquid chromatography; HPTLC, high-performance thin-layer chromatography; LC, liquid chromatography; RP-HPLC, reversed-phase HPLC; SPE, solid-phase extraction.

## 7 Mechanistic evidence and molecular pathways

Mechanistic interpretation must be preparation-, concentration-, and model-specific. A pathway should be described as directly supported only when it was measured and functionally interrogated; inference from a related metabolite, a review article, or molecular docking alone is insufficient (Figure 4).

**FIGURE 4:**
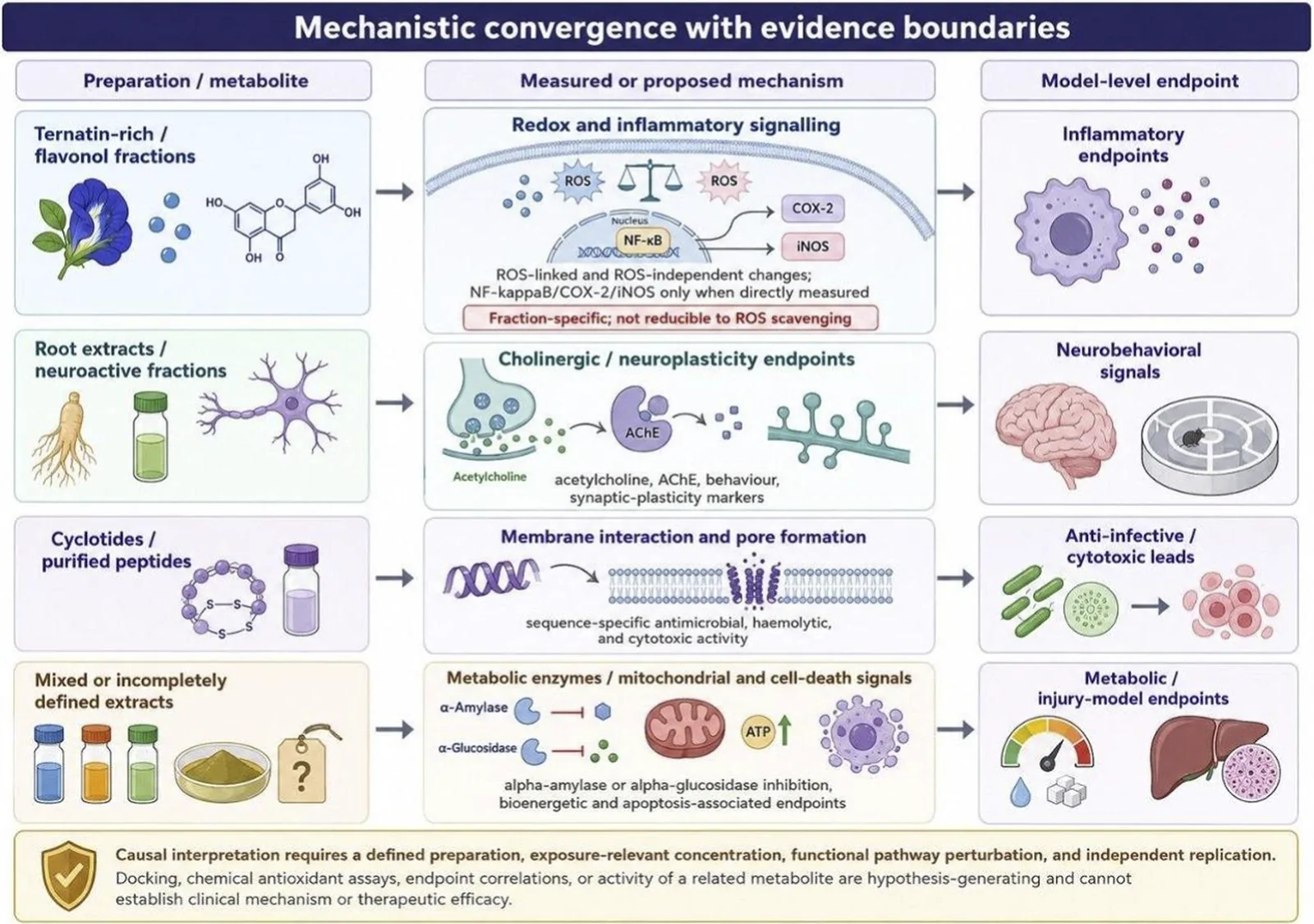
Reported or proposed mechanistic convergence and evidence limits for *Clitoria ternatea* metabolites. The schematic links major metabolite classes with redox, inflammatory, cholinergic, metabolic-enzyme, membrane, and cell-death pathways while emphasizing that the displayed outcomes are model-level signals and that evidence is preparation-, concentration-, and model-dependent. It is an original schematic created for this manuscript and finalized using Python (Pillow); no published figure was reproduced. Abbreviations: CAT, catalase; COX-2, cyclooxygenase-2; GPx, glutathione peroxidase; iNOS, inducible nitric oxide synthase; MAPK, mitogen-activated protein kinase; NF-κB, nuclear factor kappa B; PI3K/Akt, phosphoinositide 3-kinase/protein kinase B; ROS, reactive oxygen species; SOD, superoxide dismutase.

### 7.1 Chemical redox assays versus biological antioxidant evidence

DPPH, ABTS, FRAP, ORAC, and related tests are cell-free chemical or analytical assays. They can compare reducing or radical-quenching capacity under the assay conditions, but they are not *in vitro* pharmacology, do not demonstrate bioavailability, and cannot establish therapeutic antioxidant efficacy. Any half-maximal values from these tests should be labelled assay-specific EC50 or equivalent analytical endpoints rather than clinical or pharmacological IC50 values.

Biological redox evidence requires cells, tissues, animals, or humans together with appropriate controls and exposure. *C. ternatea* preparations have altered malondialdehyde, glutathione, superoxide dismutase, catalase, and related endpoints in selected models, but these effects may reflect multiple pathways and cannot be attributed solely to direct radical scavenging.

The relevant translational question is whether parent metabolites or their circulating metabolites reach the target tissue at concentrations that reproduce the experimental effect. This link is rarely established for *C. ternatea*.

### 7.2 Anti-inflammatory mechanisms: ROS-dependent and ROS-independent effects

Nair et al. (2015) demonstrated that chemically distinct petal fractions modulated LPS-stimulated macrophage inflammation through non-identical mechanisms. Ternatin-rich and quercetin-glycoside-rich fractions differed in their relationships to ROS and inflammatory mediators, showing that anti-inflammatory activity cannot be reduced to a single antioxidant mechanism.

Where directly measured, plausible downstream targets include NF-κB activation, COX-2, iNOS, nitric oxide, tumour necrosis factor-α, and interleukins. However, many *C. ternatea* publications infer these pathways from general phytochemical properties or changes in oxidative markers. Causal validation requires pathway inhibitors, knockdown or genetic models, and exposure-relevant concentrations.

Animal edema, pain, arthritis, and allergy models provide organism-level support, but their acute design and chemically undefined preparations limit extrapolation to chronic inflammatory disease.

### 7.3 Neuropharmacological mechanisms

Increased hippocampal acetylcholine, acetylcholinesterase inhibition, behavioural improvement, and hippocampal-plasticity endpoints support a cholinergic and neuroplasticity hypothesis for selected root preparations (Rai et al., 2002; Damodaran et al., 2020; Ahad et al., 2023). Cyclotides have also protected transgenic *Caenorhabditis elegans* from β-amyloid-associated toxicity, but this model does not establish human brain exposure or disease modification (Kalmankar et al., 2021).

### 7.4 Metabolic mechanisms

Enzyme inhibition, oxidative/inflammatory modulation, hepatic glycogen signalling, and postprandial physiology have been reported in different preparations. α-amylase or α-glucosidase inhibition in isolated systems is mechanistic screening; sustained glycaemic control requires oral exposure, clinically relevant dosing, and controlled human outcomes.

### 7.5 Membrane-active cyclotides and cytotoxicity

The cyclic cystine-knot confers structural stability, whereas hydrophobic and charged surface residues influence membrane binding, pore formation, antimicrobial activity, haemolysis, and cytotoxicity. These properties are peptide-specific. Crude cyclotide-rich fractions and purified sequences should not be discussed interchangeably, and normal-cell selectivity is essential before anticancer or anti-infective translation (Nguyen et al., 2011; 2016; Oguis et al., 2019).

### 7.6 Limits of mechanistic evidence

Across domains, the main deficiencies are incomplete chemical definition, concentrations that may exceed achievable exposure, reliance on endpoint correlations, limited use of functional pathway validation, and scarce replication. Molecular docking and network pharmacology may prioritize targets but remain predictive until confirmed biochemically and in exposure-relevant biological systems.

## 8 Bioavailability, pharmacokinetics, and formulation relevance

### 8.1 Ternatin absorption and metabolism

Ternatins are chemically stable pigments relative to many non-acylated anthocyanins, but stability in a food matrix does not imply high systemic bioavailability. After a single oral dose of 0.0527 mmol/kg in rats, nine intact ternatins and preternatin A3 were detectable in plasma within 15 min; total ternatin Cmax was 0.141 ± 0.035 µM, confirming oral absorption but modest systemic exposure (Ichiyanagi et al., 2021). A 2024 digestion/*in vivo* study likewise found partial anthocyanin preservation alongside extensive transformation (Yu et al., 2024). No human study has yet quantified intact ternatin pharmacokinetics or the contribution of ternatin-derived metabolites. Human anthocyanin tracer work from other sources indicates that parent anthocyanins represent only a small fraction of total exposure because degradation, microbial metabolism, methylation, glucuronidation, and sulfation generate numerous circulating metabolites (Czank et al., 2013).

### 8.2 Exposure considerations for cyclotides and other non-anthocyanin metabolites

Cyclotide proteolytic stability does not establish oral absorption or systemic exposure. Gastrointestinal recovery, epithelial permeability, parent-peptide pharmacokinetics, tissue distribution, and immunogenicity remain insufficiently characterized for C. ternatea cyclotides. Tolerability of a customary flower beverage cannot be extrapolated to purified peptides or concentrated seed and root preparations (Oguis et al., 2019).

Flavonol glycosides, phenolic acids, triterpenoids, and sterols have distinct absorption and metabolic pathways. Product development should therefore select preparation-specific marker metabolites and quantify relevant parent and circulating metabolites rather than relying on total phenolic content or extract mass alone (Czank et al., 2013).

### 8.3 Formulation and delivery systems

Microencapsulation can improve colour stability, handling, and process tolerance, while nanoemulgel and related topical systems can improve dispersion and product properties (Ab Rashid et al., 2021; Sapiun et al., 2022; Multisona et al., 2023; Sari et al., 2025). These are formulation achievements, not evidence of enhanced systemic exposure or clinical efficacy. Future work should quantify parent and metabolite pharmacokinetics, tissue distribution, dermal penetration where relevant, and formulation-specific safety.

## 9 Translational potential and clinical development

Translation should proceed by defined product rather than by species name. A traditional flower infusion, standardized oral extract, food colourant, topical nanoformulation, purified cyclotide, and butelase-enabled engineered peptide differ in composition, route, exposure, safety requirements, and regulatory pathway (Table 6).

**TABLE 6:** Translational status of *Clitoria ternatea* products and formulations.

Product/application	Defined preparation	Principal markers	Current evidence level	Supported use	Key limitation or regulatory position
Customary flower beverage	Measured dried flowers or infusion	Ternatins; flavonol glycosides	Dietary history; small acute human studies	Food/beverage and exploratory physiological research	No chronic efficacy, PK, or long-term safety package
Standardized flower extract	Marker-quantified aqueous/hydroalcoholic extract	Ternatins plus flavonols	Preclinical and small human studies	Candidate for staged nutraceutical/botanical research	Requires replicated dose-response, PK, DDI, safety, and trials
Microencapsulated pigment	Spray-dried ternatin-rich material	Protected anthocyanins	Formulation/food stability	Colour and process stability	Improved stability does not prove bioavailability or efficacy
Topical nanoemulgel	Characterized topical dispersion	Flower metabolites plus excipients	Physicochemical characterization	Prototype topical/cosmetic delivery	Dermal penetration, local PK, efficacy, irritation, and sensitization needed
Purified cyclotide	Sequence-defined peptide	Specific cliotide/Cter peptide	*In vitro* and early *in vivo* lead research	Peptide scaffold or lead discovery	Sequence-specific selectivity, PK, haemolysis, and immunogenicity required
Butelase-1 platform	Purified ligase or engineered system	Asx-specific ligase activity	Strong biochemical biotechnology evidence	Peptide macrocyclization and manufacturing tool	Not evidence for oral efficacy of a crude plant preparation
Natural food colourant	Food-grade flower pigment preparation	Ternatin profile and degradation products	Substantial chemistry/application evidence	Colouring of beverages and foods	Purity, stability, contaminants, dietary exposure, and local regulations apply
Intelligent packaging	Anthocyanin-containing polymer film	pH-responsive pigments	Materials and food-packaging studies	Freshness indicator and active packaging research	Migration, toxicology, shelf-life, and food-contact approval required
Cosmetic product	Topical flower extract/formulation	Anthocyanins; flavonols	Laboratory/formulation evidence	Colourant or cosmetic prototype	Clinical cosmetic efficacy and sensitization data are limited

Abbreviations: DDI, drug-drug interaction; PK, pharmacokinetics.

### 9.1 Human evidence and clinical interpretation

Current human studies are small and heterogeneous. Acute crossover trials support short-term physiological effects of flower preparations, while the retrospectively registered pulmonary-tuberculosis adjunctive study (NCT06794502) and the 2026 diabetic-distress study provide exploratory clinical signals (Chusak et al., 2018; Thilavech et al., 2021; Jassanti et al., 2025; Mani et al., 2026). None establishes a disease-treatment indication, and no preparation has a sufficiently replicated dose-response, pharmacokinetic, safety, and efficacy package for routine clinical recommendation.

### 9.2 Product development, commercialization, and regulatory boundaries

Food colourants and customary beverages can be developed around identity, purity, stability, dietary exposure, and permitted claims. A botanical drug requires batch-standardized composition, nonclinical pharmacology, pharmacokinetics, repeat-dose and reproductive safety, interaction testing, dose finding, and adequately powered trials. Isolated cyclotides require conventional peptide-drug evaluation, including sequence-specific potency, selectivity, bioavailability, immunogenicity, and manufacturing controls. A target product profile should predefine the route, intended population and claim, marker-normalized dose, release specifications, and the exposure and safety package required; otherwise, evidence from beverages, extracts, and purified peptides remains non-combinable.

Functional foods, intelligent packaging, and cosmetics are commercially plausible applications, but chemical antioxidant capacity or pigment incorporation should not be presented as therapeutic efficacy. Claims must remain proportionate to the evidence and intended regulatory category.

## 10 Safety, immunogenicity, and botanical-drug interaction risks

Historical dietary use documents exposure to customary flower preparations, but it is not sufficient evidence for the safety of concentrated extracts, roots, seeds, purified cyclotides, repeated medicinal use, or vulnerable populations. Safety must therefore be assessed for the exact preparation, dose, duration, and route (Table 7).

**TABLE 7:** Safety and interaction risk matrix for *Clitoria ternatea* products.

Exposure or risk	Current evidence	Unresolved concern	Required next step
Customary flower beverage	Historical food use and short acute human exposure; the 2022 EFSA supporting publication records safety objections to the EU traditional-food notification for dried flowers	Preparation-specific chronic exposure, cyclotide content, vulnerable populations, contaminants, and marker dose	Longer studies with standardized preparation and adverse-event monitoring
Concentrated flower/root extract	High-dose animal histology signal at 2,000 mg/kg after 14 days	90-day/chronic, reproductive, genotoxic, and carcinogenic safety	OECD-aligned repeat-dose and reproductive-developmental programmes
Cyclotide-rich fraction or isolated peptide	Stable membrane-active peptides with experimental activity	Haemolysis, normal-cell selectivity, complement, antibodies, cytokine release	Sequence-specific PK, toxicology, and immunogenicity package
Metabolic enzymes and transporters	Docking and a cell-line rhodamine-transport signal for P-gp; no human DDI and no adequate preparation-specific CYP/UGT/transporter dataset	Clinical relevance is unknown; CYP, UGT, P-gp, BCRP, and OATP effects remain theoretical/testing priorities	Exposure-relevant *in vitro* screening followed by clinical DDI study if warranted
Concomitant antidiabetic/TB/CNS therapy	Small co-medication studies without dedicated pharmacokinetic or interaction endpoints	Potential pharmacodynamic or pharmacokinetic interactions remain theoretical	Quantify drug and botanical-marker exposure and prospectively monitor interactions

Abbreviations: BCRP, breast cancer resistance protein; CNS, central nervous system; CYP, cytochrome P450; DDI, drug-drug interaction; OATP, organic-anion transporting polypeptide; OECD, Organisation for Economic Co-operation and Development; P-gp, P-glycoprotein; PK, pharmacokinetics; TB, tuberculosis; UGT, uridine 5-prime-diphospho-glucuronosyltransferase.

In its 2022 supporting publication on the notification of dried *C. ternatea* flowers as a traditional food from a third country, EFSA recorded safety objections because the available evidence did not exclude risk, particularly in relation to cyclotides with reported haemolytic, cytotoxic, immune, or uterine effects and limitations in the documented history of safe use (EFSA, 2022). This conclusion applies to the notified dried-flower material and intended exposure; it reinforces that historical food use should not be generalized to concentrated extracts, chronic medicinal dosing, or purified constituents.

### 10.1 Acute and repeated-dose toxicity

A 14-day mouse study reported no mortality at 500–1,000 mg/kg but liver and kidney histopathological changes at 2000 mg/kg (Khatib and Tofrizal, 2024). This identifies a high-dose safety signal but does not establish a no-observed-adverse-effect level for chronic human use. Ninety-day and longer repeat-dose studies with exposure and organ-toxicity assessment remain necessary.

### 10.2 Reproductive, genotoxic, and long-term safety

Robust fertility, embryo-fetal development, peri/postnatal, genotoxicity, carcinogenicity, and chronic clinical safety data are lacking. Traditional reports involving pregnancy should not be interpreted as evidence of reproductive safety. Concentrated supplements should therefore be used cautiously in pregnancy, lactation, childhood, older age, hepatic or renal impairment, and polypharmacy until preparation-specific evidence is available.

### 10.3 Cyclotide selectivity and immunogenicity

Cyclotides are structurally stable and membrane-active. Their pharmaceutical promise is accompanied by sequence-specific risks of haemolysis, normal-cell cytotoxicity, complement activation, cytokine release, antibody formation, or hypersensitivity. These endpoints are not adequately characterized for repeated mucosal or systemic exposure and cannot be inferred from the tolerability of a flower beverage.

### 10.4 Specific botanical-drug interaction risks

Clinically relevant botanical–drug interactions have not been demonstrated in humans. Margret et al. (2019) reported molecular docking and a cell-line rhodamine-transport signal consistent with P-glycoprotein modulation; these preclinical findings do not establish transporter inhibition, altered drug exposure, or a clinical interaction in humans. No preparation-specific CYP inhibition or induction dataset adequate for human interaction inference was identified. CYP3A4, CYP2C9, CYP2D6, UGT, P-glycoprotein, BCRP, and OATP effects are therefore prospective testing priorities rather than established clinical risks; standardized preparations should be screened at exposure-relevant concentrations. Time-dependent inhibition and induction should be distinguished from nonspecific assay interference.

### 10.5 Pharmacodynamic and clinical co-medication risks

Possible additive pharmacodynamic effects with antidiabetic or antihypertensive drugs, antiplatelet or anticoagulant therapy, and central-nervous-system medicines remain theoretical and warrant prospective monitoring rather than being described as demonstrated interactions. The tuberculosis adjunctive study and the diabetic-distress study did not include dedicated pharmacokinetic or interaction endpoints. Until such studies are available, clinical neutrality cannot be assumed, but clinically significant interactions should not be asserted.

## 11 Translational roadmap and research priorities

The revision applies the four essential domains of phytopharmacological assessment: pharmacology, composition, bioavailability, and safety. Progress depends on linking these domains for a defined product rather than accumulating additional unstandardized activity reports.

1. Define and authenticate the product: specify plant part, cultivar, route, processing, drug-to-extract ratio, voucher, fingerprint, quantified markers, contaminants, and stability.
2. Establish credible pharmacology and exposure: use disease-relevant models, negative and positive controls, randomization, blinding, dose-response, parent/metabolite pharmacokinetics, tissue exposure, and pharmacokinetic-pharmacodynamic analysis.
3. Stage safety and clinical development: complete repeat-dose, reproductive, genotoxic, immunogenicity, and interaction studies before dose-finding and adequately powered randomized trials; keep food, wellness, cosmetic, and therapeutic claims distinct.

Near-term priorities are a marker-standardized flower preparation for dietary or botanical research and sequence-defined cyclotides or butelase-enabled peptides for conventional peptide-development pathways. These routes should not be conflated.

## 12 Conclusion

*Clitoria ternatea* is chemically and biotechnologically important, but its evidence base is uneven. Ternatins are strong flower markers and useful pigments; cyclotides and butelase-1 are compelling peptide-discovery and engineering tools; and several preclinical domains show reproducible biological signals. These strengths do not resolve the central limitations of incomplete extract characterization, overinterpretation of chemical antioxidant assays, heterogeneous animal models, scarce exposure data, and limited human evidence.

The most defensible conclusions are preparation- and evidence-specific. Acute human studies support short-term physiological effects of flower preparations, not treatment of diabetes or dyslipidaemia. Exploratory adjunctive studies in tuberculosis and diabetic distress require independent replication. Direct evidence for PCOS/PCOD was not identified, and the unsupported section was removed. Anti-inflammatory activity is mechanistically heterogeneous and cannot be reduced to ROS scavenging alone.

A credible next phase requires authenticated and chemically standardized products, rigorous controls and dose-response design, exposure-matched mechanisms, full pharmacokinetic and interaction assessment, long-term and reproductive safety, peptide immunogenicity evaluation, and adequately powered randomized trials. Applying these standards will allow *C. ternatea* to be evaluated as a family of defined products rather than as a universally therapeutic species.
