# Supplementary material for: Clitoria ternatea as a phytopharmacological resource: plant metabolites, mechanistic evidence, translational prospects, and safety considerations
Source: Front Pharmacol. 2026 Oct 16;17:1922024. doi: 10.3389/fphar.2026.1922024 (PMC13719572; doi:10.3389/fphar.2026.1922024)
Supplement: Supplementary file 1 [file Table1.doc]

# Supplementary Methods

# S1. Review design

This article was conducted as a structured critical narrative review. Its purpose was to appraise a heterogeneous natural-product literature according to botanical identity, preparation chemistry, study design, exposure, bioavailability, safety, and translational relevance. It was not registered as a systematic review and no meta-analysis was attempted because plant parts, extraction methods, chemical definition, models, doses, outcomes, and study quality were too heterogeneous for valid quantitative pooling.

Searches covered database inception through 8 August 2026. Primary studies were prioritized for specific pharmacological, mechanistic, clinical, pharmacokinetic, and toxicological claims. Reviews were used for historical positioning, extraction background, and citation chaining, but not as the sole support for a study-specific efficacy claim when a primary report was identifiable.

# S2. Information sources and database-specific searches

| **Source** | **Search string or approach** |
| --- | --- |
| PubMed/MEDLINE — core query | (("Clitoria ternatea"[Title/Abstract] OR "butterfly pea"[Title/Abstract] OR "blue pea"[Title/Abstract]) AND (phytochem*[Title/Abstract] OR metabolite*[Title/Abstract] OR anthocyanin*[Title/Abstract] OR ternatin*[Title/Abstract] OR cyclotide*[Title/Abstract] OR butelase[Title/Abstract] OR extraction[Title/Abstract] OR pharmacolog*[Title/Abstract] OR mechanis*[Title/Abstract] OR bioavailab*[Title/Abstract] OR pharmacokinetic*[Title/Abstract] OR formulation*[Title/Abstract] OR toxic*[Title/Abstract] OR safety[Title/Abstract] OR interaction*[Title/Abstract] OR clinical[Title/Abstract])) |
| PubMed/MEDLINE — recent update | (("Clitoria ternatea"[Title/Abstract] OR "butterfly pea"[Title/Abstract]) AND (2024:2026[pdat])) |
| PubMed/MEDLINE — cyclotide/butelase supplement | (("Clitoria ternatea"[Title/Abstract] OR "butterfly pea"[Title/Abstract] OR butelase[Title/Abstract]) AND (cyclotide*[Title/Abstract] OR "albumin-1"[Title/Abstract] OR precursor*[Title/Abstract] OR ligase[Title/Abstract] OR peptide*[Title/Abstract])) |
| Scopus | TITLE-ABS-KEY("Clitoria ternatea" OR "butterfly pea" OR "blue pea") AND TITLE-ABS-KEY(phytochem* OR metabolite* OR anthocyanin* OR ternatin* OR cyclotide* OR butelase OR extraction OR pharmacolog* OR mechanism* OR bioavailab* OR pharmacokinetic* OR formulation* OR toxic* OR safety OR interaction* OR clinical) AND PUBYEAR < 2027 |
| ScienceDirect | "Clitoria ternatea" AND (phytochemistry OR metabolites OR extraction OR pharmacology OR mechanism OR bioavailability OR pharmacokinetics OR formulation OR safety OR toxicity OR clinical) |
| Google Scholar — targeted verification | Exact-title and author searches for landmark or recent records; combinations of "Clitoria ternatea" with anthocyanin bioavailability, cyclotide genomics, butelase-1, UHPLC-QTOF-HRMS, neuroprotection, diabetic distress, tuberculosis, nanoemulgel, CYP, transporter, toxicity, and safety. |
| Citation chaining | Reference lists of Mukherjee et al. (2008), Oguis et al. (2019), Jeyaraj et al. (2021), and Gamage et al. (2021) were screened. Forward citation searching and publisher/DOI/PubMed record checks were used to identify primary updates and verify bibliographic metadata. |

# S3. Eligibility criteria

## Included evidence:

- Peer-reviewed primary studies directly examining authenticated or identifiable Clitoria ternatea material, an isolated C. ternatea metabolite, or a defined peptide/enzyme derived from the species.
- Chemical/analytical, in silico, isolated-target, cell-based, animal, and human studies relevant to composition, extraction, pharmacology, mechanisms, bioavailability, formulation, safety, or interactions.
- Foundational reviews and methodological/guideline papers needed to position prior syntheses or apply natural-product quality standards.
- English-language records for which sufficient bibliographic and methodological information could be evaluated.

## Excluded or not used as claim-defining evidence:

- Records concerning unrelated species, commercial products with unresolvable botanical identity, or preparations whose relationship to C. ternatea could not be established.
- Duplicate records, inaccessible citations with insufficient metadata, non-peer-reviewed promotional claims, and unsourced statements.
- Purely agronomic or food-technology studies without a relevant composition, exposure, pharmacology, safety, or translational question.
- Chemical antioxidant tests used as proof of therapeutic efficacy; such studies were retained only for analytical or extraction-related interpretation.
- In silico predictions used as confirmation of efficacy; these were treated only as hypothesis-generating evidence.

# S4. Evidence hierarchy

| **Evidence stratum** | **Typical evidence** | **Permitted interpretation / principal limitation** |
| --- | --- | --- |
| Traditional/contextual | Documented historical or customary use | Generates hypotheses and exposure context; does not establish efficacy or chronic safety. |
| Chemical/analytical | DPPH, ABTS, FRAP, ORAC, pigment stability, reducing power, chelation | Characterizes chemistry or extraction performance; does not establish pharmacological efficacy. |
| In silico | Docking, network pharmacology, ADMET prediction | Prioritizes targets or metabolites; requires experimental validation. |
| Isolated target/cell | Enzyme inhibition, receptor/target assay, cultured cells | Provides mechanistic or cytotoxic signals; physiological exposure and whole-organism relevance must be demonstrated. |
| Animal | Acute or chronic in vivo models | Provides proof of concept and limited safety information; model relevance, bias control, PK, and human-equivalent exposure determine confidence. |
| Human | Acute crossover, exploratory comparative, controlled clinical studies | Highest direct relevance, but confidence depends on randomization, blinding, sample size, defined preparation, duration, clinical endpoints, and adverse-event assessment. |

# S5. Data extraction and qualitative appraisal

| **Domain** | **Information extracted / appraisal question** |
| --- | --- |
| Botanical identity | Accepted name/authority, plant part, cultivar or flower colour, location/date, authenticator, voucher or defined commercial source. |
| Preparation | Fresh/dried state, processing, extraction solvent/process, drug-to-extract ratio, yield, fractionation, storage, and formulation. |
| Chemical definition | Fingerprint method, quantified marker(s), purity for isolated metabolites, and whether the tested material can be reproduced. |
| Model and controls | Chemical assay, in silico model, target/cell system, animal model, or human design; positive/negative/vehicle controls; disease relevance. |
| Dose and exposure | Concentration or dose, route, duration, dose-response, minimal active level when reported, and relationship to plausible systemic or local exposure. |
| Bias control | Randomization, blinding, allocation concealment, sample-size justification, replicate structure, prespecified endpoints, and statistical reporting. |
| Outcomes | Magnitude and direction of effect, clinical versus surrogate endpoint, selectivity, reproducibility, and contradictory or null findings. |
| Safety | Adverse events, organ toxicity, repeated-dose exposure, reproductive/genotoxic evidence, immunogenicity, and interaction assessment. |
| Translational relevance | Defined product, PK/PD linkage, formulation, dose standardization, comparator, regulatory category, and evidence needed for the next development stage. |

# S6. Synthesis and handling of uncertainty

Studies were synthesized narratively by preparation and evidence domain. Conflicting results were not averaged across chemically different materials. Missing dose, control, duration, authentication, or marker data were recorded as limitations rather than inferred. Evidence was downgraded when the claimed therapeutic conclusion exceeded the model, when concentrations lacked exposure plausibility, when the preparation was poorly defined, or when independent replication was absent.

The literature search and reference list were updated to 8 August 2026. No numerical PRISMA flow is supplied because the original search was not prospectively managed as a registered systematic review and exact deduplicated screening counts were not preserved. This limitation is disclosed to avoid false precision.

# S7. Supplementary botanical-authentication note

Microscopy is retained only as a pharmacognostic quality-control aid. Representative diagnostic findings include a dorsiventral leaf, predominantly paracytic stomata, covering trichomes, collateral vascular tissue, lignified fibres, calcium oxalate crystals, and starch grains in powdered material. These observations should be used with an authenticated reference and cannot substitute for voucher documentation, source and processing metadata, chemical fingerprinting, marker quantification, and contaminant testing.

# S8. Abbreviations

ABTS, 2,2-prime-azino-bis(3-ethylbenzothiazoline-6-sulfonic acid); AChE, acetylcholinesterase; ADMET, absorption, distribution, metabolism, excretion, and toxicity; BCRP, breast cancer resistance protein; COX-2, cyclooxygenase-2; CYP, cytochrome P450; DDI, drug-drug interaction; DPPH, 2,2-diphenyl-1-picrylhydrazyl; FRAP, ferric reducing antioxidant power; HPLC, high-performance liquid chromatography; HPTLC, high-performance thin-layer chromatography; iNOS, inducible nitric oxide synthase; LC-MS/MS, liquid chromatography-tandem mass spectrometry; NF-kappaB, nuclear factor kappa B; NMR, nuclear magnetic resonance; OATP, organic-anion transporting polypeptide; ORAC, oxygen radical absorbance capacity; P-gp, P-glycoprotein; PK, pharmacokinetics; ROS, reactive oxygen species; UHPLC-QTOF-HRMS, ultra-high-performance liquid chromatography-quadrupole time-of-flight high-resolution mass spectrometry; UGT, uridine 5-prime-diphospho-glucuronosyltransferase.

**Verification and non-inference rule.** Bibliographic metadata were checked against PubMed, primary publisher records, DOI landing pages, or official registries where available. Missing methodological details were not inferred. Searches of ClinicalTrials.gov used “Clitoria ternatea”, “butterfly pea”, and “blue pea”; the pulmonary-tuberculosis study was matched to NCT06794502.
